# Does gender moderate the association between socioeconomic status and health? Results from an observational study in persons with spinal cord injury living in Morocco

**DOI:** 10.3389/fresc.2023.1108214

**Published:** 2023-04-04

**Authors:** Regula Limacher, Abderrazak Hajjioui, Maryam Fourtassi, Christine Fekete

**Affiliations:** ^1^ Swiss Paraplegic Research, Guido A. Zäch Institute, Nottwil, Switzerland; ^2^ Department of Health Sciences and Medicine, University of Lucerne, Lucerne, Switzerland; ^3^ Clinical Neuroscience Laboratory, Faculty of Medicine and Pharmacy, Sidi Mohamed Ben Abdallah University, Fez, Morocco; ^4^ Department of Physical and Rehabilitation Medicine, Hassan II University Hospital, Fez, Morocco; ^5^ Laboratory of Life and Health Sciences, Faculty of Medicine and Pharmacy of Tangier, Abdelmalek Essaadi University, Tangier, Morocco; ^6^ Work Mastery, Corporate Health Consulting, Lucerne, Switzerland

**Keywords:** disability, health inequalites, socioeconomic status, spinal cord injury, moderation, gender, Morocco

## Abstract

**Background:**

Socioeconomic status (SES) and gender are well-known social determinants of health. However, their impact on health in populations with physical disabilities in low-resource countries is still lacking. Therefore, the objective of this study was to investigate associations of individual SES with health and the moderating effect of gender on this association in a Moroccan population with a physical disability, namely spinal cord injury.

**Methods:**

Cross-sectional survey data from 385 participants with spinal cord injury living in Morocco were analyzed. SES was operationalized by education level, household income, financial hardship, and subjective social status. Health indicators included secondary conditions, pain, vitality, quality of life, and general health. Associations between SES and health indicators were investigated using linear and logistic regressions. To test the potential moderation of gender, interaction terms between SES and gender were introduced in regression models.

**Results:**

Financial hardship and lower subjective social status were associated with poorer health outcomes in four out of five indicators in the total sample. In contrast, education and income were inconsistently associated with health. Overall, gender did not moderate the association between SES and health, except that educational inequalities in general health were more pronounced in women, and the observation of a trend for a stronger negative effect of subjective social status on men's than woman's health (*p* > 0.05).

**Conclusion:**

This study revealed that subjective indicators of SES negatively impact on health, whereas evidence for the moderating role of gender in this association was weak. These findings underline the importance to reduce social marginalization and poverty in populations with disabilities in low-resource countries to reduce their double burden of living with a disability and encountering social disadvantages through low SES.

## Introduction

Inequalities in health among groups with different socioeconomic status (SES) are among the most robust findings in socio-epidemiological research, existing with varying extent in both, within and between countries (1–3). Although economic, social, and health indicators in low-resource countries have improved in recent years, socially determined health inequalities may even have increased (4–6). Indeed, economic growth in Morocco has never been so strong as is has been since the early 2000s (7). However, the country is enduring serious difficulties in linking prosperity and social cohesion (7). Despite the marked reduction of poverty, Morocco has experienced persistent, if not greater, income inequalities and the ratio of the richest 10% to the poorest 10% is extremely high, indicating the presence of large social inequalities (6, 7). With only about two-third of the population being granted health insurance (8), healthcare is for many Moroccan people not affordable. Appropriate research is needed to narrow the health gap between different SES groups (5, 9, 10). Evidence on health inequalities in low-resource countries remains limited to the general population, and little is known about people living with health conditions (11–13). Research among this vulnerable group is often challenging and limited due to the widespread lack of resources and infrastructure for data collection.

The Moroccan spinal cord injury (MorSCI) cohort study collected data on people with spinal cord injury (SCI) in Morocco as part of an international community survey (8). SCI is a chronic health condition of varying degrees of complexity and severity. The traumatic or non-traumatic injury of the spinal cord leads to a complete or partial loss of sensory and motor function below the lesion level, and those affected are often severely physically impaired and suffer from secondary conditions (14, 15). Some studies have already documented social inequalities in the context of SCI in Western countries (11, 13, 16, 17), while evidence for low-resource countries is lacking. In less developed countries, people with disabilities are often marginalized and more likely to experience social exclusion and discrimination (18–21). Stigma and negative stereotypes and attitudes towards socially excluded groups can affect their health and create or even reinforce health inequalities (2, 10). To inform tailored interventions and health policies, it is of interest to investigate social inequalities in health in the context of SCI in Morocco.

In the study of health inequalities, it is of growing importance to go beyond the traditional SES indicators education, income, or occupation and examine how SES affects health in a more proximal way (22). Literature suggests that more subjective parameters, such as financial hardship (11, 23, 24) and subjective social status (SSS) (25–28) are important predictors for health beyond the traditional SES indicators, reflecting one's day-to-day lived experience. This study therefore includes a comprehensive set of SES indicators, including education, household income, financial hardship, and SSS. To gain a comprehensive picture of participants' health, a broad range of health indicators was used in this study. Along with quality of life (QoL) and general health as rather general assessments, emphasis is placed on physical health, which is measured by secondary conditions, pain intensity, and vitality.

Besides the main effect of SES on health, it is likely that gender moderates the association between individual SES and health. A moderating effect is present if the effect of the independent variable on the dependent variable depends on the value of a third variable, the so-called moderator variable (29). In this case, the effect of SES on health may differ depending on one's gender as another determinant of health (30). Gender differences in health outcomes are observed across the lifespan and contribute to inequalities in morbidity and mortality (31, 32). Social, cultural, economic, and biological factors have a more substantial negative effect on women's than on men's health (31, 32). The gender gap in Morocco has been reduced substantially over the past 30 years as the Gender Inequality Index dropped from 0.74 in 1990 to 0.43 in 2021 (the lower the number, the lower gender inequalities; average all countries worldwide in 2021: 0.46) (33). Still, gender disparities in Morocco are observable in different socioeconomic indicators. More specifically, women report lower school enrolment rates, lower literacy rates, lower labor income and a lower share of non-agricultural wage labor, which may lead to poorer overall health (6).

In summary, social inequalities in health in persons with SCI have not been studied in low-resource countries and so far, no research in the context of SCI has ever investigated whether health inequalities were moderated by gender. This original and novel study thus aims at expanding the current understanding on the interplay between SES, gender, and health in persons with disabilities from low-resource countries.

**Figure 1:**
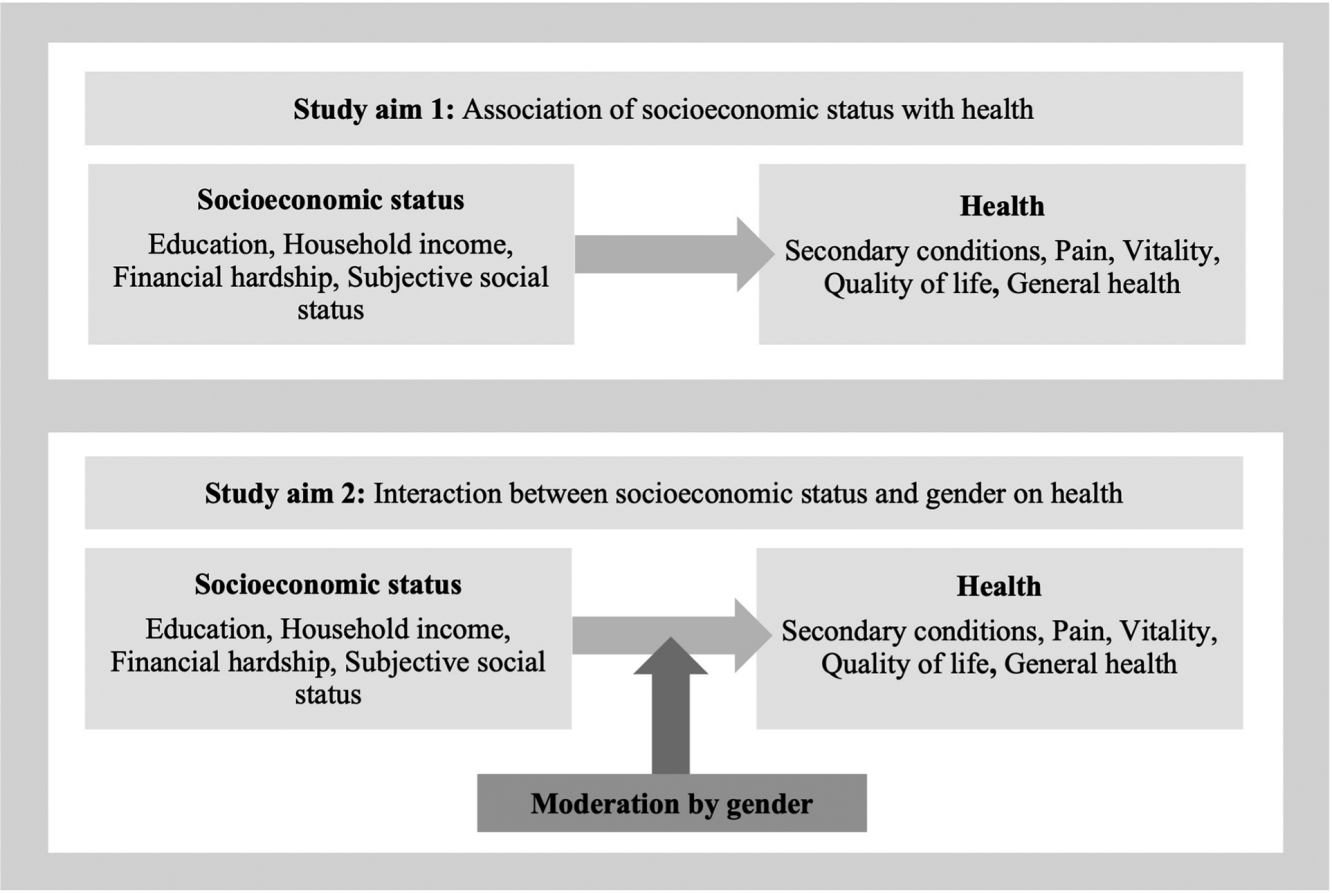
Analytical framework for the study.

In light of these substantial research gaps, the aims of this study are twofold (see Figure 1):

(1) To investigate the association of SES (education, household income, financial hardship, and SSS) with health (secondary conditions, pain, vitality, QoL, and general health) for the total population and stratified by gender.
(2) To examine the potentially moderating role of gender in the association between SES and health.

It is hypothesized that I) lower SES is associated with poorer health outcomes, and II) that social inequalities in health are more pronounced in women than in men.

## Materials and methods

### Design, setting and sample

Cross-sectional data from 385 participants of the population-based community survey of the MorSCI Cohort Study were analyzed (12). The MorSCI survey is part of the International Spinal Cord Injury (InSCI) Survey, a multi-country survey on people with SCI living in a community (8, 34, 35). Ethics committee approval was obtained prior to recruitment, and informed consent was provided by all participants after being informed of the study objectives (8, 34). The MorSCI sample was recruited from 20 institutions (8 rehabilitation facilities; 6 emergency and general hospitals; 3 patient organizations; 3 governmental agencies) using convenience sampling methods (8). The survey was mainly conducted by telephone or face-to-face interview while very few responded to the questionnaire online (8). The 125-item questionnaire was developed by the InSCI study group and was translated from English to Arabic (8, 34, 35). The MorSCI community survey was conducted between June 2017 and December 2018 (8). Persons with traumatic or non-traumatic SCI over 18 years old were included in the study, while people with congenital etiologies of SCI, neurodegenerative disorders, or Guillain Barré syndrome were excluded (8, 34).

### Measures

#### Socioeconomic status

*Education* was assessed according to the International Standard Classification of Education as the highest completed level of education, combining general and vocational education (36). The categories post-secondary, short tertiary, bachelor or equivalent, and master or equivalent were combined to higher education as only few people indicated those education levels. Lower secondary and higher secondary education were merged into the category secondary education, thus resulting in four categories: no schooling, primary education, secondary education, and higher education.

*Household income* was used to assess the participants' income situation. It was measured by the total household income, weighted by the number of adults and children living in the household. The item for household income was based on the Model Disability Survey (Item H1017) (37). The response options were based on the guidelines of the European Social Survey and were categorized into ranges of deciles of current household income in Morocco (38). The criteria of the Organization for Economic Co-operation and Development (OECD) were applied for weighting (1.0 for the first adult; 0.5 for each additional adult over 14 years; 0.3 for children) (39, 40). Household income was classified into distribution-based quartiles for analyses.

*Financial hardship* was assessed with a 4-point Likert scaled item asking how much a problematic financial situation has influenced participation in society in the last four weeks. Response options included “not applicable”, “no influence”, “made my life a little harder”, and “made my life a lot harder”. The first two categories were combined into one, resulting in the three-categorical variable for the analysis: no financial hardship, some financial hardship, and massive financial hardship.

*SSS.* The Mac Arthur Scale of Subjective Social Status was used to assess the participants' SSS in society, visualized with a ten-step ladder (41, 42). Participants were asked to place themselves on the ladder representing the social hierarchy relative to other people living in Morocco. Due to a floor effect in the distribution of this variable, a categorical variable representing low (1,2), middle (3–5), and high (>5) SSS was created for analysis.

#### Health outcomes

*Secondary conditions.* The Spinal Cord Injury Secondary Condition Scale (SCI-SCS) was used to capture the prevalence and severity of secondary conditions in the last three months (43). The scale was modified by not recording the items on heterotopic bone ossification and diabetes mellitus but adding sleep problems. Since pain was measured as a separate health indicator, two items on pain were omitted. Therefore, the SCI-SCS used in this study included 13 items. The original 4-point Likert scale of the SCI-SCS was modified to a 5-point ordinal scale ranging from 1 (no problem) to 5 (extreme problem) following the Model Disability Survey (37). For analysis, the scale was recoded from 0 (no problem) to 4 (extreme problem), yielding a sum score ranging from 0 to 52, with a higher score indicating higher burden of secondary conditions.

*Pain* was assessed by asking participants to rate their pain as its worst in the last week on a numerical scale ranging from 0 (no pain) to 10 (pain as bad as you can imagine). The item was derived from the Brief Pain Inventory (44–46) and was used as a continuous variable in analysis.

*Vitality* was measured using the 4-item vitality index of the 36-item Short Form Health Survey (SF-36) (47). The items included the following questions: “How much of the time during the last 4 weeks did you feel full of life?” “…did you have a lot of energy?” “…did you feel worn out?” “…did you feel tired?”. The response options ranged from “all of the time” to “none of the time” and were coded with a 5-point scale from 1 to 5. Items three and four were recoded to 1, 2, 3.5, 5 and 6, and items one and two reversed to 6, 5, 3.5, 2 and 1 according to the SF-36 Manual and Interpretation Guide (47). The raw scale scores were transformed to a 0–100 scale, with a higher score indicating higher vitality.

*Quality of life* was assessed with the World Health Organization Quality of Life Assessment-5 (WHOQoL-5) (48). It consists of 5 items and enquires the overall quality of life and satisfaction with health, daily activities, relationships, and living conditions over the last 14 days (48). Each item was rated on a 5-point Likert scale from 0 (very dissatisfied) to 4 (very satisfied) and resulted in a global sum score ranging from 0 to 20, with a higher score indicating better QoL.

*General health* was assessed by single item “General health” of the 12-item Short Form Health Survey, rating their health in general as “excellent”, “very good”, “good”, “fair” or “poor” (49, 50). General Health was analyzed as a dichotomous variable, whereby “excellent”, “very good” and “good” were categorized as good health, and “fair” and “poor” were captured as poor health.

#### Control variables

Sociodemographic data (age, gender), lesion characteristics, and mobility status were included as potential confounders in the multivariable analysis, given their impact on health (14, 51). Lesion characteristics considered the injury level (paraplegia vs. tetraplegia), lesion completeness (complete vs. incomplete), time since injury, and etiology (traumatic vs. non-traumatic) (15, 52). Mobility status was included as a categorical variable and included the categories walking independently, manual wheelchair or supervision walking, and total assistance or electric wheelchair.

### Statistical analysis

Statistical analysis was conducted using Stata version 16.0 for Mac (College Station, TX, USA). The distribution of all SES indicators, health outcomes, and control variables was described. The analysis was conducted for the total sample and stratified by gender. The data set is comprehensive, with only one missing data point reported for the variable vitality. To examine gender differences in SES and health variables, chi-square tests were conducted for categorical and binary variables and t-tests for continuous variables.

To investigate associations between SES and health, linear regressions were used for continuous outcomes secondary conditions, pain, vitality and QoL and logistic regression was used for the binary outcome general health. Two sets of regression models were calculated. First, health outcomes were separately regressed on each SES variable in an unadjusted model (Model 1; 5 models per outcome). Then, models were adjusted for all control variables and all SES indicators (Model 2; 1 model per outcome). The two models were performed for the total sample and stratified by gender. Coefficients and odds ratios (ORs), together with the 95% confidence intervals (CI), are reported for continuous and binary outcomes, respectively. Likelihood ratio tests were used to calculate the *p*-value for associations under investigation. Sensitivity analysis with a third model adjusted for all SES indicators was performed.

To test moderation, we followed the state-of-the-art approach to include an interaction term between the moderator variable (i.e., gender) and the SES indicators into regression models for each health outcome (53, 54). The interaction term was composed of gender and the individual SES indicators in order to test the interaction of these two variables in the association between SES and health (i.e., in respective regression models), with gender as the moderating factor being examined. The models were adjusted for all control variables. *P*-values were obtained from likelihood ratio tests using the *contrast* command. *P*-values of interaction terms <0.05 indicate a significant interaction or moderation of the variable gender. In other words, significant interaction terms indicate, that social inequalities in health differ between males and females.

## Results

### Sample characteristics and gender differences

Basic characteristics of the total sample and stratified by gender are displayed in Table 1. The majority of the sample was male (72.5%), the mean age was 38.7 years. The mean years since injury were 7.1 years, and most reported a traumatic cause of the SCI. Regarding education, 17.9% have never attended school, 21.3% have completed primary school, 39.2% had secondary education, and 21.6% had higher education. Two-thirds of the participants reported having massive financial hardship. 37.4% classified their social status as low and just over one out of ten considered themselves to be in the upper half of the social hierarchy. With regard to their health, participants reported on average 15.5 points on the secondary conditions scale (range 0–52), 4.2 on the pain intensity scale (range 0–10), 56.8 on the vitality scale (range 0–100) and 10.8 on the QoL scale (range 0–20) Less than one in four reported their general health to be good.

**Table 1:** Basic characteristics of the MorSCI population.

[Missing values]	*N* (%) or mean (SD); *median (IQR)*			*p*-value
	**Total**	**Male**	**Female**	
*N*	385 (100)	279 (72.47)	106 (27.53)	
**Sociodemographic**				
Age in years	38.68 (13.22*); 37* (*28–47*)	37.79 (12.70); *35* (*28–46*)	41.01 (14.29); *39 (28–50)*	0.033
**Lesion characteristics**				
Paraplegia	285 (74.03)	205 (73.48)	80 (75.47)	0.690
Tetraplegia	100 (25.97)	74 (26.52)	26 (24.53)	
Complete	172 (44.68)	138 (49.46)	34 (32.08)	0.002
Incomplete	213 (55.32)	141 (50.54)	72 (67.92)	
Traumatic etiology	298 (77.40)	233 (83.51)	65 (61.32)	0.000
Non-traumatic etiology	87 (22.60)	46 (16.49)	41 (38.68)	
Years since injury	7.06 (8.43); *4 (2–9)*	6.93 (8.08); *4 (2–8)*	7.40 (9.33); *3 (2–9)*	0.630
**Mobility**				0.018
Total assistance or electric wheelchair	147 (38.18)	106 (37.99)	41 (38.68)	
Manual wheelchair or supervision walking	155 (40.26)	122 (43.73)	33 (31.13)	
Walking independently with or without aids	83 (21.56)	51 (18.28)	32 (30.19)	
**Education level**				<0.001
No schooling	69 (17.92)	41 (14.70)	28 (26.42)	
Primary education	82 (21.30)	68 (24.37)	14 (13.21	
Secondary education	151 (39.22)	120 (43.01)	31 (29.25)	
Higher education	83 (21.56)	50 (17.92)	33 (31.13)	
**Household income**				0.892
Lowest quartile	85 (22.08)	64 (22.94)	21 (19.81)	
2nd lowest quartile	104 (27.01)	76 (27.24)	28 (26.42)	
2nd highest quartile	98 (25.45)	69 (24.73)	29 (27.36)	
Highest quartile	98 (25.45)	70 (25.09)	28 (26.42)	
**Financial hardship**				0.442
Massive financial hardship	255 (66.23)	188 (67.38)	67 (63.21)	
Some financial hardship	103 (26.75)	70 (25.09)	33 (31.13)	
No financial hardship	27 (7.01)	21 (7.53)	6 (5.66)	
**Subjective Social Status** (1–10)				0.610
Low (1–2)	144 (37.40)	104 (37.28)	40 (37.74)	
Middle (3–5)	196 (50.91)	145 (51.97)	51 (48.11)	
High (6–10)	45 (11.69)	30 (10.75)	15 (14.15)	
**Health indicators**				
Secondary conditions (0–52)	15.51 (7.67); *15 (10–20)*	15.67 (7.69); *16 (10–20)*	15.09 (7.62); *14 (10–21)*	0.508
Pain (0–10)	4.23 (3.19*); 4 (1–7)*	3.98 (3.24); *4 (0–6)*	4.89 (2.96); *5 (3–7)*	0.012
Vitality (0–100) [1]	56.75 (19.59); *58.33 (41.67–70.83)*	59.13 (19.39); *58.33 (45.83–70.83)*	50.51 (18.79); *52.08 (35.42–64.58)*	<0.001
Quality of life (0–20)	10.82 (3.65); *11 (9–14)*	10.92 (3.44); *11 (9–14)*	10.56 (4.15); *10.5 (9–13)*	0.382
Good health	88 (22.86)	66 (23.66)	22 (20.75)	0.545
Poor health	297 (77.14)	213 (76.34)	16 (79.25)	

*p*-value from chi-square test for binary and categorical variables or t-test for continuous variables.

*IQR*, interquartile range; *N*, number of individuals; *SD*, standard deviation; *MorSCI*, moroccan spinal cord injury community survey.

Significant gender differences were found for age, lesion characteristics, mobility, education, pain, and vitality. Men were younger, more often completely paralyzed and more frequently reported a traumatic cause of SCI. The proportion of participants who could walk was higher for women than for men. The gender difference in education was ambiguous. More women reported no schooling, however, the proportion of women in the highest level of education was higher than for men. Regarding self-reported health, men reported on average almost a 1-point lower pain intensity and 10% higher vitality than women.

### Study aim 1: association of socioeconomic status with health

#### Results for the total sample

Results of bivariate and multivariable analyses of the total sample are presented in Table 2. Perceived financial hardship was associated with all health outcomes, i.e., more secondary conditions, increased pain intensity, reduced vitality, lower QoL, and higher odds for poor general health in model 1. Results were approved in model 2 (except for general health, *p* > 0.05). Association with SSS were significant for four out of five health outcomes (secondary conditions, vitality, QoL, and general health) in the unadjusted and adjusted model. Education and income were inconsistently associated with health and only three relevant associations were detected, whereby two of them were against the hypothesized direction (higher education—more secondary conditions; higher income—decreased pain intensity; higher income—decreased vitality).

**Table 2:** Unadjusted and adjusted associations of socioeconomic status with health indicators for the total sample.

	Secondary conditions 0-52		Pain 0-10		Vitality 0-100		Quality of life 0-20		General health Good/Poor	
	Model 1	Model 2	Model 1	Model 2	Model 1	Model 2	Model 1	Model 2	Model 1	Model 2
	ß (95% CI)		ß (95% CI)		ß (95% CI)		ß (95% CI)		OR (95% CI)	
**Education level**										
No schooling	Ref	Ref	Ref	Ref	Ref	Ref	Ref	Ref	Ref	Ref
Primary education	−0.11 (−2.57–2.36)	0.92 (−1.42–3.25)	−0.09 (−1.11–0.93)	0.51 (−0.51–1.54)	4.57 (−1.65–10.99)	−0.49 (−6.61–5.63)	0.11 (−1.07–1.28)	−0.52 (−1.63–0.59)	1.16 (0.54–2.49)	1.01 (0.43–2.40)
Secondary education	0.23 (−1.97–2.42)	1.13 (−1.10–3.37)	−0.51 (−1.42–0.40)	0.13 (−0.85–1.12)	4.32 (−1.30–9.95)	−0.56 (−6.41–5.29)	0.09 (−0.96–1.14)	−0.76 (−1.82–0.31)	0.97 (0.48–1.93)	0.80 (0.35–1.87)
Higher education	1.13 (−1.33–3.60)	3.42 (0.90–5.94)	0.13 (–0.89–1.15)	1.02 (−0.08–2.13)	4.15 (−2.15–10.45)	–0.85 (–7.43–5.74)	0.24 (−0.93–1.42)	−1.34 (−2.54–−0.14)	1.22 (0.57–2.60)	0.69 (0.27–1.78)
***p-value***	***0.723***	***0.041***	***0.436***	***0.160***	***0.423***	***0.996***	***0.982***	***0.181***	***0.879***	***0.815***
R^2^	0.004	0.218	0.007	0.128	0.007	0.185	0.001	0.216	0.002	0.142
**Household income**										
Lowest quartile	Ref	Ref	Ref	Ref	Ref	Ref	Ref	Ref	Ref	Ref
2nd lowest quartile	1.09 (−1.12–3.30)	0.80 (−1.22–2.83)	0.01 (−0.90–0.93)	−0.05 (−0.94–0.84)	–2.89 (–8.55–2.78)	−3.48 (−8.78–1.82)	−0.41 (−1.44–0.63)	−0.55 (−1.51–0.42)	0.63 (0.29–1.33)	0.55 (0.24–1.26)
2nd highest quartile	−0.20 (−2.44–2.03)	0.75 (−1.38–2.87)	−0.42 (−1.35–0.51)	−0.38 (−1.32–0.55)	–1.93 (–7.67–3.81)	−5.79 (−11.35− −0.23)	0.66 (−0.39–1.71)	−0.21 (−1.23–0.80)	1.49 (0.75–2.94)	0.89 (0.41–1.94)
Highest quartile	0.74 (−1.49–2.98)	1.78 (−0.56–4.13)	−0.46 (−1.39–0.47)	−0.38 (−1.41–0.65)	–2.87 (–8.61–2.87)	−9.26 (−15.38–−3.14)	1.10 (0.05–2.15)	−0.15 (−1.27–0.97)	1.42 (0.71–2.80)	0.65 (0.27–1.57)
***p-value***	***0.600***	***0.516***	***0.598***	***0.806***	***0.735***	***0.028***	***0.017***	***0.720***	***0.067***	***0.445***
R^2^	0.005	0.218	0.005	0.128	0.003	0.185	0.026	0.216	0.053	0.142
**Financial hardship**										
Massive financial hardship	Ref	Ref	Ref	Ref	Ref	Ref	Ref	Ref	Ref	Ref
Some financial hardship	−3.11 (−4.83–−1.39)	−2.53 (−4.28–−0.78)	−0.38 (−1.10–0.35)	−0.33 (−1.10–0.44)	6.48 (2.05–10.91)	4.96 (0.39–9.54)	1.80 (1.00–2.61)	1.12 (0.28–1.95)	2.50 (1.47–4.23)	1.86 (1.00–3.44)
No financial hardship	−4.51 (−7.50–−1.52)	−3.57 (−6.75–−0.39)	−2.19 (−3.44–−0.93)	−1.92 (−3.32–−0.53)	11.21 (3.53–18.88)	8.44 (0.16–16.72)	3.26 (1.87–4.65)	1.76 (0.25–3.28)	4.06 (1.77–9.29)	1.89 (0.67–5.34)
***p-value***	***<0.001***	***0.007***	***0. 003***	***0.026***	***0. 001***	***0.037***	***<0.001***	***0.009***	***<0.001***	***0.124***
R^2^	0.046	0.218	0.031	0.128	0.036	0.185	0.084	0.216	0.044	0.142
**Subjective Social Status**										
Low	Ref	Ref	Ref	Ref	Ref	Ref	Ref	Ref	Ref	Ref
Middle	−1.83 (−3.47– –0.19)	−2.06 (−3.73–−0.39)	−0.32 (−1.01–0.37)	−0.19 (−0.93–0.54)	6.82 (2.68–10.96)	8.59 (4.22–12.96)	1.66 (0.91–2.42)	1.58 (0.79–2.38)	2.77 (1.53–5.03)	2.81 (1.42–5.57)
High	−3.45 (−6.00– –0.90)	−3.15 (−5.89–−0.42)	−0.44 (−1.52–0.63)	0.13 (−1.07–1.32)	13.08 (6.64–19.52)	14.57 (7.44–21.69)	3.35 (2.18–4.52)	2.88 (1.58–4.18)	4.98 (2.28–10.89)	5.07 (1.87–13.76)
***p-value***	***0.013***	***0.025***	***0.5751***	***0.767***	***<0.001***	***<0.001***	***<0.001***	***<0.001***	***<0.001***	***0.003***
R^2^	0.022	0.218	0.003	0.128	0.049	0.185	0.089	0.216	0.048	0.142

Coefficients (ß) from linear regressions and odds ratios (OR) from logistic regressions and their 95% confidence intervals (CI).

All linear trends are shown in color. Light green indicates a trend in favor of the hypothesis, with dark green being significant at *p*-value < 0.05. Light red indicates a trend against the hypothesis, with dark red being significant at *p*-value < 0.05.

*p*-values from likelihood ratio tests.

Model 1: unadjusted.

Model 2: adjusted for age, gender, lesion level, completeness of injury, years since injury, etiology, mobility, and all SES indicators.

*Ref*, reference category; *ß*, coefficient; *OR*, odds ratio; *CI*, confidence interval.

#### Results by gender

The results from the regression analysis for men and women are presented in Tables 3, 4, respectively. For men, financial hardship was associated with worse health in all outcomes under investigation but became insignificant for pain, vitality, and general health in model 2. Lower SSS was significantly associated with worse health in four (model 1) and three (model 2) health outcomes for men. The results for financial hardship and SSS generally showed similar results for women but were less linear and less robust. Only the association of SSS and general health differed from findings for men. Education was not associated with health, except that higher education was associated with higher odds of being in good general health in women (adjusted model). Income was inconsistently associated with health in any indicator, in both, men and women.

**Table 3:** Unadjusted and adjusted associations of socioeconomic status with health indicators for men.

	Secondary conditions 0–52		Pain 0–10		Vitality 0–100		Quality of life 0–20		General health Good/Poor	
	Model 1	Model 2	Model 1	Model 2	Model 1	Model 2	Model 1	Model 2	Model 1	Model 2
	ß (95% CI)		ß (95% CI)		ß (95% CI)		ß (95% CI)		OR (95% CI)	
**Education level**										
No schooling	Ref	Ref	Ref	Ref	Ref	Ref	Ref	Ref	Ref	Ref
Primary education	−0.03 (−3.04-2.98)	0.67 (−2.17–3.50)	−0.05 (−1.31–1.22)	0.31 (−0.95–1.58)	0.27 (−7.36–7.91)	−0.94 (−8.20–6.32)	−0.51 (−1.85–0.83)	−0.88 (−2.12–0.37)	0.81 (0.34–1.92)	0.81 (0.31–2.17)
Secondary education	−0.14 (−2.89–2.61)	0.75 (−2.01–3.50)	−0.53 (−1.69–0.62)	0.14 (−1.09–1.37)	−0.03 (−7.03–6.96)	−1.54 (−8.60–5.52)	−0.45 (−1.67–0.78)	−0.93 (−2.14–0.28)	0.51 (0.23–1.17)	0.49 (0.18–1.33)
Higher education	0.19 (−3.01–3.40)	2.17 (−1.04–5.38)	−0.27 (−1.61–1.08)	0.88 (−0.55–2.31)	3.26 (−4.87–11.39)	−0.48 (−8.70–7.74)	0.51 (−0.91–1.94)	−0.91 (−2.32–0.50)	1.14 (0.46–2.79)	0.77 (0.25–2.35)
***p-value***	***0.996***	***0.574***	***0.708***	***0.562***	***0.773***	***0.972***	***0.329***	***0.457***	***0.163***	***0.476***
R^2^	0.000	0.208	0.005	0.109	0.004	0.191	0.012	0.240	0.017	0.172
**Household income**										
Lowest quartile	Ref	Ref	Ref	Ref	Ref	Ref	Ref	Ref	Ref	Ref
2nd lowest quartile	1.72 (−0.85–4.29)	1.65 (−0.80–4.09)	−0.11 (−1.19–0.97)	−0.02 (−1.11–1.07)	−2.46 (−8.99–4.06)	−4.15 (−10.40–2.10)	0.03 (−1.12–1.17)	−0.06 (−1.13–1.02)	0.60 (0.25–1.44)	0.50 (0.19–1.34)
2nd highest quartile	0.23 (−2.40–2.86)	1.47 (−1.15–4.09)	−0.64 (−1.74–0.46)	−0.56 (−1.73–0.61)	−2.38 (−9.05–4.30)	−8.22 (−14.91–−1.53)	0.75 (−0.42–1.92)	−0.30 (−1.45–0.85)	1.79 (0.82–3.88)	0.93 (0.37–2.36)
Highest quartile	0.53 (−2.10–3.15)	1.29 (−1.53–4.11)	−1.08 (−2.18–0.02)	−1.02 (−2.28–0.23)	−3.78 (−10.43–2.87)	−11.09 (−18.29–−3.90)	1.22 (0.06–2.39)	−0.14 (−1.38–1.09)	1.24 (0.56–2.75)	0.47 (0.16–1.40)
***p-value***	***0.543***	***0.572***	***0.174***	***0.337***	***0.732***	***0.019***	***0.100***	***0.959***	***0.067***	***0.263***
R^2^	0.008	0.208	0.018	0.109	0.005	0.191	0.022	0.240	0.025	0.172
**Financial hardship**										
Massive financial hardship	Ref	Ref	Ref	Ref	Ref	Ref	Ref	Ref	Ref	Ref
Some financial hardship	−3.78 (−5.85–−1.72)	−3.00 (−5.14–−0.86)	−0.44 (−1.32–0.44)	−0.25 (−1.21–0.71)	6.41 (1.15–11.68)	4.31 (−1.15–9.78)	1.36 (0.44–2.27)	0.69 (−0.25–1.63)	2.00 (1.07–3.74)	1.56 (0.74–3.32)
No financial hardship	−4.38 (−7.77–−0.99)	–2.99 (–6.68–0.70)	−2.21 (−3.65–−0.76)	−1.72 (−3.37–−0.07)	11.40 (2.75–20.06)	9.81 (0.40–19.23)	3.34 (1.84–4.84)	2.18 (0.56–3.80)	3.28 (1.28–8.39)	1.74 (0.5*0*–6.06)
***p-value***	***<0.001***	***0.016***	***0.011***	***0.124***	***0.005***	***0.073***	***<0.001***	***0.024***	***0.012***	***0.444***
R^2^	0.058	0.208	0.033	0.109	0.038	0.191	0.080	0.240	0.013	0.172
**Subjective Social Status**										
Low	Ref	Ref	Ref	Ref	Ref	Ref	Ref	Ref	Ref	Ref
Middle	−1.54 (−3.47–−0.39)	−1.86 (−3.87–0.15)	−0.33 (−1.15–0.49)	−0.03 (−0.93–0.87)	5.94 (1.18–10.71)	9.24 (4.12–14.37)	1.74 (0.92–2.56)	1.84 (0.96–2.72)	2.92 (1.45–5.90)	3.41 (1.50–7.76)
High	–3.74 (−6.86–−0.63)	−2.71 (−6.08–0.65)	−0.76 (−2.09–0.56)	0.24 (−1.27–1.74)	17.06 (9.39–24.74)	19.04 (10.45–27.62)	3.83 (2.50–5.15)	3.34 (1.87–4.82)	6.71 (2.63–17.11)	8.90 (2.63–30.11)
***p-value***	***0.047***	***0.135***	***0.484***	***0.928***	***<0.001***	***<0.001***	***<0.001***	***<0.001***	***<0.001***	***0.001***
R^2^	0.022	0.208	0.005	0.109	0.068	0.191	0.120	0.240	0.061	0.172

Coefficients (ß) from linear regressions and odds ratios (OR) from logistic regressions and their 95% confidence interval (CI).

All linear trends are shown in color. Light green indicates a trend in favor of the hypothesis, with dark green being significant at *p*-value < 0.05. Light red indicates a trend against the hypothesis, with dark red being significant at *p*-value < 0.05.

*p*-values from likelihood ratio tests.

Model 1: unadjusted.

Model 2: adjusted for age, lesion level, completeness of injury, years since injury, etiology, mobility, and all SES indicators.

*Ref*, reference category; *ß*, coefficient; *OR*, odds ratio; *CI*, confidence interval.

**Table 4:** Unadjusted and adjusted associations of socioeconomic status with health indicators for women.

	Secondary conditions 0–52		Pain 0–10		Vitality 0–100		Quality of life 0–20		General health Good/Poor	
	Model 1	Model 2	Model 1	Model 2	Model 1	Model 2	Model 1	Model 2	Model 1	Model 2
	ß (95% CI)		ß (95% CI)		ß (95% CI)		ß (95% CI)		OR (95% CI)	
**Education level**										
No schooling	Ref	Ref	Ref	Ref	Ref	Ref	Ref	Ref	Ref	Ref
Primary education	−2.18 (−7.10–2.74)	−1.10 (−6.03–3.83)	0.21 (−1.72–2.15)	0.63 (−1.45–2.72)	7.74 (−4.45–19.93)	5.83 (−7.56–19.23)	1.64 (−1.05–4.33)	1.08 (−1.62–3.78)	2.27 (0.39–13.08)	1.67 (0.14–19.76)
Secondary education	0.44 (−3.48–4.36)	1.40 (−2.81–5.60)	−0.06 (−1.60–1.48)	−0.27 (−2.05–1.51)	8.16 (−1.55–17.87)	5.81 (−5.62–17.23)	1.12 (−1.02–3.27)	0.57 (−1.74–2.88)	4.58 (1.12–18.69)	11.39 (1.23–115.57)
Higher education	2.54 (−1.33–6.40)	4.35 (−0.13–8.83)	0.75 (−0.77–2.27)	1.00 (−0.90–2.90)	5.03 (−4.54–14.60)	2.63 (−9.55–14.81)	−0.19 (−2.31–1.92)	−1.56 (−4.02–0.90)	1.49 (0.32–6.87)	1.11 (0.09–13.94)
***p-value***	***0.245***	***0.111***	***0.690***	***0.388***	***0.372***	***0.701***	***0.383***	***0.114***	***0.112***	***0.025***
R^2^	0.040	0.343	0.014	0.220	0.030	0.202	0.029	0.333	0.059	0.326
**Household income**										
Lowest quartile	Ref	Ref	Ref	Ref	Ref	Ref	Ref	Ref	Ref	Ref
2nd lowest quartile	−0.64 (−5.04–3.75)	−1.51 (−5.58–2.57)	0.25 (−1.36–2.05)	-0.05 (−1.78–1.67)	−3.30 (−14.17–7.57)	−2.41 (−13.49–8.66)	−1.60 (−3.94–0.75)	−1.67 (−3.91–0.56)	0.71 (0.16–3.23)	0.99 (0.13–7.45)
2nd highest quartile	−1.29 (−5.65–3.07)	−0.45 (−4.60–3.70)	0.11 (−1.58–1.80)	0.06 (−1.70–1.81)	0.73 (−10.06–11.52)	−1.97 (−13.24–9.30)	0.40 (−1.93–2.73)	−0.46 (−2.74–1.81)	0.89 (0.21–3.79)	0.49 (0.06–3.99)
Highest quartile	1.25 (−3.14–5.64)	3.20 (−1.49–7.90)	1.10 (−0.61–2.80)	1.27 (−0.71–3.26)	0.72 (−10.15–11.59)	−3.40 (16.14–9.35)	0.76 (−1.59–3.11)	−0.24 (−2.81–2.33)	2.01 (0.52–7.75)	1.35 (0.15–11.91)
***p-value***	***0.641***	***0.188***	***0.535***	***0.468***	***0.835***	***0.957***	***0.152***	***0.431***	***0.382***	***0.737***
R^2^	0.016	0.343	0.021	0.220	0.008	0.202	0.050	0.333	0.028	0.326
**Financial hardship**										
Massive financial hardship	Ref	Ref	Ref	Ref	Ref	Ref	Ref	Ref	Ref	Ref
Some financial hardship	−1.49 (−4.69–1.71)	−1.52 (−4.93–1.90)	−0.39 (−1.64–0.85)	−0.87 (−2.32–0.58)	8.23 (0.42–16.05)	4.77 (−4.51–14.05)	2.90 (1.24–4.56)	2.16 (0.29–4.03)	4.90 (1.70–14.09)	7.49 (1.70–33.04)
No financial hardship	−5.18 (−11.60–1.23)	−4.27 (−11.12–2.59)	−1.95 (−4.45–0.54)	−2.65 (−5.55–0.25)	8.80 (−6.86–24.46)	3.46 (−15.17–22.09)	2.84 (−0.49–6.17)	0.92 (−2.84–4.68)	8.57 (1.44–50.90)	8.63 (0.57–130.41)
***p-value***	***0.226***	***0.407***	***0.284***	***0.159***	***0.088***	***0.594***	***0.002***	***0.076***	***0.004***	***0.026***
R^2^	0.028	0.343	0.024	0.220	0.046	0.202	0.114	0.333	0.003	0.326
**Subjective Social Status**										
Low	Ref	Ref	Ref	Ref	Ref	Ref	Ref	Ref	Ref	Ref
Middle	−2.66 (−5.83–0.52)	−2.81 (−6.16–0.54)	−0.35 (−1.51–0.10)	−0.28 (−1.70–1.14)	8.67 (0.91–16.43)	7.58 (−1.53–16.69)	1.44 (−0.28–3.15)	0.62 (−1.22–3.46)	2.39 (0.77–7.41)	0.83 (0.14–4.90)
High	−2.84 (−7.39–1.71)	−4.30 (−9.51–0.91)	0.07 (−1.73–1.86)	0.11 (−2.10–2.31)	6.53 (−4.60–17.65)	2.61 (−11.55–16.76)	2.41 (−0.50–4.87)	1.40 (−1.46–4.25)	2.55 (0.58–11.17)	0.38 (0.04–4.17)
***p-value***	***0.211***	***0.178***	***0.893***	***0.862***	***0.087***	***0.212***	***0.099***	***0.617***	***0.277***	***0.671***
R^2^	0.030	0.343	0.002	0.220	0.046	0.202	0.044	0.333	0.026	0.326

Coefficients (ß) from linear regressions and odds ratios (OR) from logistic regressions and their 95% confidence interval (CI).

All linear trends are shown in color. Light green indicates a trend in favor of the hypothesis, with dark green being significant at *p*-value < 0.05.

*p*-values from likelihood ratio tests.

Model 1: unadjusted.

Model 2: adjusted for age, lesion level, completeness of injury, years since injury, etiology, mobility, and all SES indicators.

*Ref*, reference category; *ß*, coefficient; *OR*, odds ratio; *CI*, confidence interval.

### Study aim 2: the moderating role of gender in the association between SES and health

The results of the interactions between SES and gender on health are displayed in Table 5. Results generally indicate that gender does not moderate the association between SES and health. However, a trend for moderation was observed for SSS (*p* > 0.05), as the impact of SSS was more pronounced for men than for women in four out of five health outcomes (secondary condition, pain, QoL, and general health). The interaction of education and gender on general health showed a significant result, indicating that the educational inequalities in women are larger than in men. No moderating effects of gender were found for income and financial hardship.

**Table 5:** Interactions of socioeconomic status and gender on health outcomes.

	Secondary conditions 0–52 ß (95% CI)	Pain 0–10 ß (95% CI)	Vitality 0–100 ß (95% CI)	QoL 0–20 ß (95% CI)	Health Good/Poor OR (95% CI)
**Education level**					
No schooling x male	Ref	Ref	Ref	Ref	Ref
Primary education x male	1.95 (−3.49–7.39)	−0.16 (−2.52–2.19)	−5.40 (−19.88–9.09)	−2.54 (−5.22–0.15)	0.22 (0.03–1.79)
Secondary education x male	−0.72 (−5.19–3.76)	−0.11 (−2.05–1.83)	−7.12 (−19.03–4.80)	−1.70 (−3.90–0.51)	0.08 (0.01–0.44)
Higher education x male	−3.26 (−7.96–1.45)	−0.88 (−2.92–1.15)	−0.73 (−13.24–11.79)	0.51 (−1.81–2.83)	0.61 (0.09–3.94)
***p-value for interaction term***	***0.246***	***0.809***	***0.579***	***0.052***	***0.008***
R^2^	0.174	0.103	0.107	0.115	0.104
**Household income**					
Lowest quartile x male	Ref	Ref	Ref	Ref	Ref
2nd lowest quartile x male	2.64 (−2.14–7.42)	0.13 (1.04)	−0.59 (−13.27–12.09)	1.71 (−0.62–4.04)	0.95 (0.15–5.92)
2nd highest quartile x male	1.83 (−2.94–6.60)	−0.21 (1.04)	−4.93 (−17.60–7.74)	0.39 (−1.93–2.72)	2.58 (0.45–14.79)
Highest quartile x male	−2.02 (−6.82–2.78)	−1.86 (1.05)	−4.77 (−17.50–7.97)	0.68 (−1.66–3.02)	0.75 (0.14–3.94)
***p-value for interaction term***	***0.170***	***0.150***	***0.782***	***0.483***	***0.439***
R^2^	0.170	0.112	0.106	0.129	0.097
**Financial hardship**					
Massive financial hardship x male	Ref	Ref	Ref	Ref	Ref
Some financial hardship x male	−1.83 (−5.39–1.74)	−0.06 (−1.61–1.48)	−1.79 (−11.19–7.60)	−1.48 (−3.20–0.24)	0.35 (0.10–1.25)
No financial hardship x male	−0.68 (−7.42–6.06)	−0.10 (−3.02–2.83)	3.18 (−14.59–20.94)	0.78 (−2.48–4.03)	0.54 (0.07–4.32)
***p-value for interaction term***	***0.602***	***0.996***	***0.856***	***0.184***	***0.266***
R^2^	0.184	0.111	0.132	0.160	0.108
**Subjective social status**					
Low SSS x male	Ref	Ref	Ref	Ref	Ref
Middle SSS x male	−0.21 (−3.68–3.26)	−0.12 (−1.64–1.39)	−2.41 (−11.36–6.54)	0.33 (−1.31–1.98)	1.26 (0.31–5.11)
High SSS x male	−1.66 (−6.81–3.49)	−0.69 (−2.93–1.55)	11.12 (−2.14–24.38)	1.39 (−1.04–3.83)	3.18 (0.50–20.09)
***p-value for interaction term***	***0.811***	***0.830***	***0.119***	***0.531***	***0.439***
R^2^	0.177	0.097	0.164	0.187	0.129

Coefficients (ß) from linear regressions and odds ratios (OR) from logistic regressions and their 95% confidence interval (CI).

All linear trends are shown in color. Light green indicates a trend in favor of the hypothesis, with dark green being significant at *p*-value < 0.05. Light red indicates a trend against the hypothesis.

*p*-values for interaction terms from likelihood ratio tests.

All models are adjusted for age, lesion level, completeness of injury, years since injury, etiology, and mobility.

*ß*, coefficient; *OR*, odds ratio; *CI*, confidence interval; *QoL*, quality of life; *SSS*, subjective social status, *Ref*, reference category.

## Discussion

This study of Moroccan men and women living with a disability indicates that subjective indicators of the SES (financial hardship and SSS) are consistently associated with health outcomes, whereas the association of education and income with health was inconsistent. Our hypothesis to find consistent health inequalities for a range of SES indicators was thus only confirmed for the more subjective indicators and less so for the traditional indicators education and income. Overall, the results did not support the moderating hypothesis stating that negative effects of socioeconomic status on health were amplified in women compared to men. Only the results of the interaction of education and gender on general health indicated educational inequalities to be larger in women than in men. A trend for moderation was observed for SSS (*p* > 0.05), as the impact of SSS on health was more pronounced for men than for women.

### SES and health

The inconsistent results of the SES indicators for the association with health raise concerns about which SES indicators are crucial for explaining socioeconomic differences in health among persons with physical disabilities. In health inequalities research, objective measures such as education or income are traditional indicators to describe the relationship between SES and health, but associations with health were not observed in our study. One possible explanation could be that people with SCI are often marginalized due to their disability in low-resource countries such as Morocco and that this social disadvantage is better reflected in the more subjective measures, such as SSS, as it has already been reported for other SCI populations (11, 55). Objective measurements of SES are subsequently not as meaningful in this population. A closer look at the results reveals that the coefficients and ORs of income and education for all health outcomes, except the relationship between income and pain, generally weaken or reverse the assumed relationship after controlling for all confounders and SES indicators. This observation suggests that in the current study, the effects attributed to education or income in a simple model are driven by other factors such as age, lesion characteristics, mobility, and other SES indicators when the model is extended to include these additional variables. Conversely, results for financial hardship and SSS remained relatively robust after adjustment and are in line with previous findings (11, 25, 28).

The sensitivity analysis (see Supplementary Tables) showed that including the SES variables, in addition to all the confounding variables mentioned, had a stronger impact on associations of income and education on health than it was observed for SSS and financial hardship. Financial hardship and SSS have been found to mediate the association of income and education with health in previous studies (11, 28, 56). However, this cannot be explained conclusively in this study. Furthermore, as the study is based on cross-sectional data, it cannot be evaluated whether the subjective measures of SES cause poorer health or result from people's health problems.

There are multiple explanations for the constant associations of SSS and financial hardship with health. SSS might reflect a person's individual SES more comprehensively than more objective measures and therefore shows associations with health even when controlled for other SES measures (27), i.e., SSS has additional explanatory power over standard objective SES indicators like education or income. Additionally, the relative comparison of oneself in society can lead to subjective psychological consequences, negatively affecting physical and mental health (57). Similarly, financial hardship can create a sense of relative deprivation and lead to a heightened stress response, negatively impacting health (58). Financial hardship may further serve as a better indicator of the individual economic burden of people with disabilities due to their increased expenditure on health care and material deprivation such as lack of goods, opportunities, and resources may be adequately addressed (11, 59). Previous findings in high-income countries support the strong impact of financial hardship on the health of people with SCI (11).

Contrary to the hypothesis, education showed a significant positive association with secondary condition after adjustment for all confounders and other SES indicators. The coefficients for income showed a similar trend but were less pronounced and insignificant. Even when counterintuitive, these results can have several explanations. The literature confirms that lower education is associated with later referral among people with chronic health conditions (58), explaining that higher educated individuals are more likely to be diagnosed with secondary conditions. In countries where inequalities in health care services exist in favor of persons with higher SES (5, 6), income and education may play an essential role in detection of secondary conditions, as it reflects a person's access to health services (16). For persons with low SES, this may result in undiagnosed secondary conditions on one hand and untreated health conditions on the other. Comparing different countries showed that people with SCI in low-resource countries are more likely to die from preventable secondary conditions, while secondary conditions are no longer the leading cause of death for people with SCI in high-income countries (52).

### Gender differences and moderation

The proportion of women and men among the study participants is representative, as the SCI prevalence is known to have a male-to-female ratio of at least 2:1 among adults (34, 52). The gender differences regarding age and lesion characteristics found in this study align with previous findings (34, 52). Women's ability to move around better and more independently may be attributed to their lower severity of the injury, as incomplete SCI preserves some sensory and motor functions below the lesion level (15). Previous results for the general Moroccan population indicate a gender difference in SES benefiting men (6, 60). The absence of these gender inequalities in SES in the present study population may be related to the general social exclusion of people affected by SCI in this country, equally valid for men and women (19, 20). The gender differences found in our study for pain (higher in females) and vitality (lower in females) are consistent with previous findings from studies conducted among populations with chronic or long-term disabilities (61, 62).

The fact that we did not observe a moderating effect of gender on the relationship between SES and health may be accompanied by methodological limitations when including interaction terms in a quantitative model. When main effects explain a large amount of the variance in an outcome, small but meaningful interactions between two variables can be difficult to detect (63). Nevertheless, a final point that needs to be discussed is the favorable but non-significant result of the moderating effect of SSS and gender on four out of five health outcomes, showing that inequalities in health are more pronounced in males than in females. This could be explained by the fact that lower SSS has an even more detrimental effect on health for men because they cannot fulfil the social norms of the gender role, e.g., by having a lower income and being unable to provide for the family.

### Strengths and limitations

This is the first study examining the moderating role of gender on the association between SES and health among physically people with a physical disability and one of the first to examine health inequalities in a low-resource country and therefore contributes to health inequalities research in the setting of disability. A major strength of this study is the comprehensive set of SES indicators and health outcomes, allowing for a detailed analysis of key drivers for health inequalities. Validated measures for most of the constructs under investigation were used and the study population is based on a community sample including people from all country regions. The data meet high-quality standards with virtually no missing data.

This study has several limitations. Although the present study helps to understand the association between SES and health, the cross-sectional nature of data precludes the determination of causality and reverse causation (i.e., that poor health caused poor SES) cannot be excluded. Additionally, health is a complex construct that is determined by various factors. Therefore, other variables not included in the analysis may be important to fully understand the relationship between SES and the selected health indicators (e.g., psychosocial resources). The generalizability of results to the total population of individuals with SCI in Morocco might be limited due to sampling bias as convenience sampling was used. This recruiting strategy includes possible selection bias as the sample was based on 20 collaborating institutions. Also, it is worthwhile mentioning that the absence of statistical significance at the traditional level of *p* < 0.05 for analysis stratified for gender might be due to low sample size, especially in females. Those results still indicate a consistent trend for inequalities in the subjective SES indicators and absence of statistical significance should be interpreted with caution. It can further not be assessed whether self-report of SES and health indicators has led to biased responses. Information on SES might be prone to social desirability bias and people with lower SES might be reluctant to report themselves as being worse off because of stigmatization. Self-reported data might also result in a bias in health outcomes. People may not be aware of certain health conditions, as disadvantaged people often have poorer health literacy and less access to health services.

## Conclusion

In this study on health inequalities in persons with a physical disability (SCI), financial hardship and lower SSS were consistently related to reduced health, whereas income and education were not associated with health. However, evidence for a moderation effect of gender was weak in our sample. Given the importance of subjective measures of SES on health, the results of this study provide evidence that the proximal social context and the lived experience of relative deprivation account most for health inequalities in this setting. These findings underline the importance to reduce social marginalization and poverty in populations with disabilities in low-resource countries to reduce their double burden of being stigmatized due to the disability and encountering social disadvantages through low SES.

## Data Availability

The original contributions presented in the study are included in the article/**Supplementary Material**, further inquiries can be directed to the corresponding author/s.
