# Supplementary material for: Does gender moderate the association between socioeconomic status and health? Results from an observational study in persons with spinal cord injury living in Morocco
Source: Front Rehabil Sci. 2023 Apr 4;4:1108214. doi: 10.3389/fresc.2023.1108214 (PMC10110871; doi:10.3389/fresc.2023.1108214)
Supplement: Supplementary file 1 [file Table1.docx]

Supplementary Material

**Does gender moderate the association between socioeconomic status and health? Results from an observational study in persons with spinal cord injury living in Morocco**

**Regula Limacher, Abderrazak Hajjioui, Maryam Fourtassi, Christine Fekete***

*** Correspondence:** Christine Fekete: christine.fekete@workmastery.ch

# Supplementary Tables

**1.1 Sensitivity analysis with and without mutual adjustment for socioeconomic indicators in the adjusted regression model for the total sample**

| Adjusted associations of socioeconomic status with health indicators for the total sample | | | | | | | | | | |
| --- | --- | --- | --- | --- | --- | --- | --- | --- | --- | --- |
|  | **Secondary conditions**  **0-52** | | **Pain**  **0-10** | | **Vitality**  **0-100** | | **Quality of life**  **0-20** | | **General health**  **Good/Poor** | |
|  | **Model 2** | **Model 3** | **Model 2** | **Model 3** | **Model 2** | **Model 3** | **Model 2** | **Model 3** | **Model 2** | **Model 3** |
|  | ß (95% CI) | | ß (95% CI) | | ß (95% CI) | | ß (95% CI) | | OR (95% CI) | |
| **Education level** | | | | | | | | | | |
| No schooling | Ref | Ref | Ref | Ref | Ref | Ref | Ref | Ref | Ref | Ref |
| Primary education | 0.44 (-1.93-2.81) | 0.92 (-1.42-3.25) | 0.40 (-0.62-1.42) | 0.51 (-0.51-1.54) | 0.97 (-5.32-7.26) | -0.49 (-6.61-5.63) | -0.16 (-1.33-1.01) | -0.52 (-1.63-0.59) | 1.13 (0.50-2.57) | 1.01 (0.43-2.40) |
| Secondary education | -0.04 (-2.24-2.16) | 1.13 (-1.10-3.37) | -0.17 (-1.12-0.78) | 0.13 (-0.85-1.12) | 2.46 (-3.39-8.31) | -0.56 (-6.41-5.29) | 0.22 (-0.87-1.31) | -0.76 (-1.82-0.31) | 1.31 (0.60-2.84) | 0.80 (0.35-1.87) |
| Higher education | 1.84 (-0.54-4.21) | 3.42 (0.90-5.94) | 0.53 (-0.50-1.55) | 1.02 (-0.08-2.13) | 3.19 (-3.12-9.51) | -0.85 (-7.43-5.74) | 0.14 (-1.04-1.32) | -1.34 (-2.54- -0.14) | 1.39 (0.61-3.16) | 0.69 (0.27-1.78) |
| ***p-value*** | ***0.266*** | ***0.041*** | ***0.328*** | ***0.160*** | ***0.738*** | ***0.996*** | ***0.892*** | ***0.181*** | ***0.854*** | ***0.815*** |
| R^2^ | 0.165 | 0.218 | 0.101 | 0.128 | 0.103 | 0.185 | 0.096 | 0.216 | 0.074 | 0.142 |
| **Household income** | | | | | | | | | | |
| Lowest quartile | Ref | Ref | Ref | Ref | Ref | Ref | Ref | Ref | Ref | Ref |
| 2^nd^ lowest quartile | 0.89 (-1.18-2.96) | 0.80 (-1.22-2.83) | 0.06 (-0.83-0.96) | -0.05 (-0.94-0.84) | -3.26 (-8.74-2.22) | -3.48 (-8.78-1.82) | -0.53 (-1.54-0.48) | -0.55 (-1.51-0.42) | 0.59 (0.27-1.30) | 0.55 (0.24-1.26) |
| 2^nd^ highest quartile | -0.16 (-2.25-1.93) | 0.75 (-1.38-2.87) | -0.40 (-1.30-0.50) | -0.38 (-1.32-0.55) | -1.77 (-7.30-3.77) | -5.79 (-11.35- -0.23) | 0.51 (-0.51-1.52) | -0.21 (-1.23-0.80) | 1.40 (0.68-2.85) | 0.89 (0.41-1.94) |
| Highest quartile | 0.28 (-1.83-2.38) | 1.78 (-0.56-4.13) | -0.53 (-1.44-0.38) | -0.38 (-1.41-0.65) | -1.20 (-6.78-4.38) | -9.26 (-15.38- -3.14) | 1.15 (0.12-2.17) | -0.15 (-1.27-0.97) | 1.47 (0.71-3.02) | 0.65 (0.27-1.57) |
| ***p-value*** | ***0.744*** | ***0.516*** | ***0.466*** | ***0.806*** | ***0.699*** | ***0.028*** | ***0.007*** | ***0.720*** | ***0.074*** | ***0.445*** |
| R^2^ | 0.159 | 0.218 | 0.099 | 0.128 | 0.103 | 0.185 | 0.123 | 0.216 | 0.090 | 0.142 |
| **Financial hardship** | | | | | | | | | | |
| Massive financial hardship | Ref | Ref | Ref | Ref | Ref | Ref | Ref | Ref | Ref | Ref |
| Some financial hardship | -2.56 (-4.20- -0.92) | -2.53 (-4.28- -0.78) | -0.35 (-1.05-0.36) | -0.33 (-1.10-0.44) | 6.60 (2.28-10.92) | 4.96 (0.39-9.54) | 1.65 (0.85-2.44) | 1.12 (0.28-1.95) | 2.41 (1.38-4.20) | 1.86 (1.00-3.44) |
| No financial hardship | -3.11 (-6.01- -0.21) | -3.57 (-6.75- -0.39) | -1.75 (-3.01- -0.50) | -1.92 (-3.32- -0.53) | 10.41 (2.77-18.04) | 8.44 (0.16-16.72) | 2.62 (1.22-4.03) | 1.76 (0.25-3.28) | 2.72 (1.13-6.58) | 1.89 (0.67-5.34) |
| ***p-value*** | ***0.003*** | ***0.007*** | ***0.022*** | ***0.026*** | ***0.001*** | ***0.037*** | ***< 0.001*** | ***0.009*** | ***0.003*** | ***0.124*** |
| R^2^ | 0.182 | 0.218 | 0.111 | 0.128 | 0.132 | 0.185 | 0.152 | 0.216 | 0.101 | 0.142 |
| **Subjective Social Status** | | | | | | | | | | |
| Low | Ref | Ref | Ref | Ref | Ref | Ref | Ref | Ref | Ref | Ref |
| Middle | -1.94 (-3.47- -0.41) | -2.06 (-3.73- -0.39) | -0.38 (-1.04-0.29) | -0.19 (-0.93-0.54) | 7.45 (3.48-11.43) | 8.59 (4.22-12.96) | 1.74 (1.03-2.47) | 1.58 (0.79-2.38) | 3.02 (1.62-5.62) | 2.81 (1.42-5.57) |
| High | -3.04 (-5.43- -0.65) | -3.15 (-5.89- -0.42) | -0.38 (-1.42-0.66) | 0.13 (-1.07-1.32) | 13.85 (7.66-20.05) | 14.57 (7.44-21.69) | 3.33 (2.20-4.46) | 2.88 (1.58-4.18) | 5.81 (2.51-13.44) | 5.07 (1.87-13.76) |
| ***p-value*** | ***0.011*** | ***0.025*** | ***0.508*** | ***0.767*** | ***< 0.001*** | ***< 0.001*** | ***< 0.001*** | ***< 0.001*** | ***<0.001*** | ***0.003*** |
| R^2^ | 0.176 | 0.218 | 0.096 | 0.128 | 0.154 | 0.185 | 0.185 | 0.216 | 0.125 | 0.142 |
| Coefficients (ß) from linear regressions and odds ratios (OR) from logistic regressions and their 95% confidence interval (CI).  All linear trends are shown in color. Light green indicates a trend in favor of the hypothesis, with dark green being significant at p-value < 0. 05.Light red indicates a trend against the hypothesis, with dark red being significant at p-value < 0.05.  p-values from likelihood ratio tests  Model 2: adjusted for age, gender, lesion level, completeness of injury, years since injury, etiology, and mobility  Model 3: adjusted for age, gender, lesion level, completeness of injury, years since injury, etiology, mobility, and all SES indicators (displayed as Model 2 in Table 2)  Unadjusted results are displayed in Model 1 of Table 2.  Abbreviation: *Ref* Reference category | | | | | | | | | | |

**1.2 Sensitivity analysis with and without mutual adjustment for socioeconomic indicators in the adjusted regression model for men**

| Adjusted associations of socioeconomic status with health indicators for men | | | | | | | | | | |
| --- | --- | --- | --- | --- | --- | --- | --- | --- | --- | --- |
|  | **Secondary conditions**  **0-52** | | **Pain**  **0-10** | | **Vitality**  **0-100** | | **Quality of life**  **0-20** | | **General health**  **Good/Poor** | |
|  | **Model 2** | **Model 3** | **Model 2** | **Model 3** | **Model 2** | **Model 3** | **Model 2** | **Model 3** | **Model 2** | **Model 3** |
|  | ß (95% CI) | | ß (95% CI) | | ß (95% CI) | | ß (95% CI) | | OR (95% CI) | |
| **Education level** | | | | | | | | | | |
| No schooling | Ref | Ref | Ref | Ref | Ref | Ref | Ref | Ref | Ref | Ref |
| Primary education | 0.57 (-2.30-3.43) | 0.67 (-2.17-3.50) | 0.31 (-0.95-1.57) | 0.31 (-0.95-1.58) | -0.54 (-8.12-7.05) | -0.94 (-8.20-6.32) | -0.76 (-2.09-0.56) | -0.88 (-2.12-0.37) | 0.75 (0.30-1.89) | 0.81 (0.31-2.17) |
| Secondary education | -0.34 (-3.08-2.40) | 0.75 (-2.01-3.50) | -0.16 (-1.37-1.04) | 0.14 (-1.09-1.37) | 0.16 (-7.10-7.42) | -1.54 (-8.60-5.52) | -0.35 (-1.62-0.92) | -0.93 (-2.14-0.28) | 0.68 (0.28-1.68) | 0.49 (0.18-1.33) |
| Higher education | 0.60 (-2.48-3.68) | 2.17 (-1.04-5.38) | 0.24 (-1.12-1.59) | 0.88 (-0.55-2.31) | 3.02 (-5.13-11.16) | -0.48 (-8.70-7.74) | 0.42 (-1.00-1.84) | -0.91 (-2.32-0.50) | 1.36 (0.52-3.57) | 0.77 (0.25-2.35) |
| ***p-value*** | ***0.820*** | ***0.574*** | ***0.771*** | ***0.562*** | ***0.772*** | ***0.972*** | ***0.277*** | ***0.457*** | ***0.372*** | ***0.476*** |
| R^2^ | 0.151 | 0.208 | 0.072 | 0.109 | 0.077 | 0.191 | 0.097 | 0.240 | 0.090 | 0.172 |
| **Household income** | | | | | | | | | | |
| Lowest quartile | Ref | Ref | Ref | Ref | Ref | Ref | Ref | Ref | Ref | Ref |
| 2^nd^ lowest quartile | 1.75 (-0.71-4.20) | 1.65 (-0.80-4.09) | 0.13 (-0.95-1.21) | -0.02 (-1.11-1.07) | -3.71 (-10.21-2.80) | -4.15 (-10.40-2.10) | -0.02 (-1.15-1.11) | -0.06 (-1.13-1.02) | 0.61 (0.24-1.53) | 0.50 (0.19-1.34) |
| 2^nd^ highest quartile | 0.50 (-1.99-2.99) | 1.47 (-1.15-4.09) | -0.45 (-1.54-0.64) | -0.56 (-1.73-0.61) | -2.98 (-9.58-3.61) | -8.22 (-14.91- -1.53) | 0.66 (-0.48-1.80) | -0.30 (-1.45-0.85) | 1.79 (0.79-4.10) | 0.93 (0.37-2.36) |
| Highest quartile | -0.36 (-2.85-2.14) | 1.29 (-1.53-4.11) | -1.09 (-2.18-0.01) | -1.02 (-2.28-0.23) | -1.85 (-8.47-4.76) | -11.09 (-18.29- -3.90) | 1.48 (0.34-2.63) | -0.14 (-1.38-1.09) | 1.32 (0.56-3.12) | 0.47 (0.16-1.40) |
| ***p-value*** | ***0.341*** | ***0.572*** | ***0.107*** | ***0.337*** | ***0.704*** | ***0.019*** | ***0.028*** | ***0.959*** | ***0.087*** | ***0.263*** |
| R^2^ | 0.159 | 0.208 | 0.089 | 0.109 | 0.078 | 0.191 | 0.114 | 0.240 | 0.103 | 0.172 |
| **Financial hardship** | | | | | | | | | | |
| Massive financial hardship | Ref | Ref | Ref | Ref | Ref | Ref | Ref | Ref | Ref | Ref |
| Some financial hardship | -3.24 (-5.24- -1.25) | -3.00 (-5.14- -0.86) | -0.38 (-1.26-0.51) | -0.25 (-1.21-0.71) | 5.99 (0.73-11.25) | 4.31 (-1.15-9.78) | 1.27 (0.34-2.18) | 0.69 (-0.25-1.63) | 1.90 (0.98-3.69) | 1.56 (0.74-3.32) |
| No financial hardship | -3.35 (-6.59- -0.01) | -2.99 (-6.68-0.70) | -1.92 (-3.40- -0.44) | -1.72 (-3.37- -0.07) | 12.67 (3.87-21.47) | 9.81 (0.40-19.23) | 3.21 (1.69-4.74) | 2.18 (0.56-3.80) | 2.48 (0.89-6.89) | 1.74 (0.5*0*-6.06) |
| ***p-value*** | ***0.002*** | ***0.016*** | ***0.038*** | ***0.124*** | ***0.004*** | ***0.073*** | ***< 0.001*** | ***0.024*** | ***0.067*** | ***0.444*** |
| R^2^ | 0.185 | 0.208 | 0.090 | 0.109 | 0.110 | 0.191 | 0.151 | 0.240 | 0.098 | 0.172 |
| **Subjective Social Status** | | | | | | | | | | |
| Low | Ref | Ref | Ref | Ref | Ref | Ref | Ref | Ref | Ref | Ref |
| Middle | -2.03 (-3.85- -0.22) | -1.86 (-3.87-0.15) | -0.41 (-1.22-0.39) | -0.03 (-0.93-0.87) | 6.91 (2.26-11.57) | 9.24 (4.12-14.37) | 1.88 (1.09-2.68) | 1.84 (0.96-2.72) | 3.21 (1.54-6.68) | 3.41 (1.50-7.76) |
| High | -3.40 (-6.35- -0.44) | -2.71 (-6.08-0.65) | -0.57 (-1.89-0.74) | 0.24 (-1.27-1.74) | 17.78 (10.22-25.35) | 19.04 (10.45-27.62) | 3.90 (2.61-5.19) | 3.34 (1.87-4.82) | 8.96 (3.19-25.13) | 8.90 (2.63-30.11) |
| ***p-value*** | ***0.026*** | ***0.135*** | ***0.523*** | ***0.928*** | ***< 0.001*** | ***< 0.001*** | ***< 0.001*** | ***< 0.001*** | ***<0.001*** | ***0.001*** |
| R^2^ | 0.171 | 0.208 | 0.072 | 0.109 | 0.147 | 0.191 | 0.210 | 0.240 | 0.149 | 0.172 |
| Coefficients (ß) from linear regressions and odds ratios (OR) from logistic regressions and their 95% confidence interval (CI).  All linear trends are shown in color. Light green indicates a trend in favor of the hypothesis, with dark green being significant at p-value < 0.05. Light red indicates a trend against the hypothesis, with dark red being significant at p-value < 0.05.  p-values from likelihood ratio tests  Model 2: adjusted for age, lesion level, completeness of injury, years since injury, etiology, and mobility  Model 3: adjusted for age, lesion level, completeness of injury, years since injury, etiology, mobility, and all SES indicators (displayed as Model 2 in Table 3)  Unadjusted results are displayed in Model 1 of Table 3.  Abbreviation: *Ref* Reference category | | | | | | | | | | |

**1.3 Sensitivity analysis with and without mutual adjustment for socioeconomic indicators in the adjusted regression model for women**

| Adjusted associations of socioeconomic status with health indicators for women | | | | | | | | | | |
| --- | --- | --- | --- | --- | --- | --- | --- | --- | --- | --- |
|  | **Secondary conditions**  **0-52** | | **Pain**  **0-10** | | **Vitality**  **0-100** | | **Quality of life**  **0-20** | | **General health**  **Good/Poor** | |
|  | **Model 2** | **Model 3** | **Model 2** | **Model 3** | **Model 2** | **Model 3** | **Model 2** | **Model 3** | **Model 2** | **Model 3** |
|  | ß (95% CI) | | ß (95% CI) | | ß (95% CI) | | ß (95% CI) | | OR (95% CI) | |
| **Education level** | | | | | | | | | | |
| No schooling | Ref | Ref | Ref | Ref | Ref | Ref | Ref | Ref | Ref | Ref |
| Primary education | -2.11 (-6.87-2.65) | -1.10 (-6.03-3.83) | 0.22 (-1.76-2.20) | 0.63 (-1.45-2.72) | 9.55 (-3.13-22.23) | 5.83 (-7.56-19.23) | 2.33 (-0.34-5.01) | 1.08 (-1.62-3.78) | 3.54 (0.45-27-93) | 1.67 (0.14-19.76) |
| Secondary education | 0.30 (-3.52-4.11) | 1.40 (-2.81-5.60) | -0.31 (-1.89-1.27) | -0.27 (-2.05-1.51) | 9.11 (-1.05-19-26) | 5.81 (-5.62-17.23) | 1.90 (-0.24-4.05) | 0.57 (-1.74-2.88) | 13.15 (2.11-81.87) | 11.39 (1.23-115.57) |
| Higher education | 3.70 (-0.20-7.60) | 4.35 (-0.13-8.83) | 1.00 (-0.62-2.61) | 1.00 (-0.90-2.90) | 5.55 (-4.84-15.94) | 2.63 (-9.55-14.81) | -0.01 (-2.21-2.18) | -1.56 (-4.02-0.90) | 2.07 (0.31-13.62) | 1.11 (0.09-13.94) |
| ***p-value*** | ***0.063*** | ***0.111*** | ***0.347*** | ***0.388*** | ***0.283*** | ***0.701*** | ***0.085*** | ***0.114*** | ***0.021*** | ***0.025*** |
| R^2^ | 0.272 | 0.343 | 0.170 | 0.220 | 0.150 | 0.202 | 0.224 | 0.333 | 0.223 | 0.326 |
| **Household income** | | | | | | | | | | |
| Lowest quartile | Ref | Ref | Ref | Ref | Ref | Ref | Ref | Ref | Ref | Ref |
| 2^nd^ lowest quartile | -1.30 (-5.46-2.85) | -1.51 (-5.58-2.57) | -0.02 (-1.71-1.69) | -0.05 (-1.78-1.67) | -3.10 (-14.15-7.94) | -2.41 (-13.49-8.66) | -1.60 (-3.88-0.74) | -1.67 (-3.91-0.56) | 0.73 (0.13-4.06) | 0.99 (0.13-7.45) |
| 2^nd^ highest quartile | -1.05 (-5.19-3.09) | -0.45 (-4.60-3.70) | -0.09 (-1.79-1.61) | 0.06 (-1.70-1.81) | 0.17 (-10.84-11.18) | -1.97 (-13.24-9.30) | 0.31 (-1.99-2.62) | -0.46 (-2.74-1.81) | 0.81 (0.15-4.39) | 0.49 (0.06-3.99) |
| Highest quartile | 2.10 (-2.13-6.33) | 3.20 (-1.49-7.90) | 0.89 (-0.84-2.63) | 1.27 (-0.71-3.26) | 1.10 (-10.14-12.34) | -3.40 (16.14-9.35) | 0.68 (-1.67-3.04) | -0.24 (-2.81-2.33) | 2.11 (0.43-10.45) | 1.35 (0.15-11.91) |
| ***p-value*** | ***0.271*** | ***0.188*** | ***0.558*** | ***0.468*** | ***0.855*** | ***0.957*** | ***0.163*** | ***0.431*** | ***0.428*** | ***0.737*** |
| R^2^ | 0.246 | 0.343 | 0.159 | 0.220 | 0.123 | 0.202 | 0.211 | 0.333 | 0.141 | 0.326 |
| **Financial hardship** | | | | | | | | | | |
| Massive financial hardship | Ref | Ref | Ref | Ref | Ref | Ref | Ref | Ref | Ref | Ref |
| Some financial hardship | -1.53 (-4.59-1.52) | -1.52 (-4.93-1.90) | -0.41 (-1.65-0.83) | -0.87 (-2.32-0.58) | 7.25 (-0.63-15.14) | 4.77 (-4.51-14.05) | 2.73 (1.11-4.36) | 2.16 (0.29-4.03) | 5.47 (1.69-17.72) | 7.49 (1.70-33.04) |
| No financial hardship | -1.54 (-7.83-4.75) | -4.27 (-11.12-2.59) | -1.19 (-3.75-1.36) | -2.65 (-5.55-0.25) | 5.64 (-10.61-21.88) | 3.46 (-15.17-22.09) | 1.46 (-1.89-4.81) | 0.92 (-2.84-4.68) | 5.73 (0.77-42.86) | 8.63 (0.57-130.41) |
| ***p-value*** | ***0.576*** | ***0.407*** | ***0.571*** | ***0.159*** | ***0.179*** | ***0.594*** | ***0.005*** | ***0.076*** | ***0.012*** | ***0.026*** |
| R^2^ | 0.224 | 0.343 | 0.151 | 0.220 | 0.147 | 0.202 | 0.255 | 0.333 | 0.205 | 0.326 |
| **Subjective Social Status** | | | | | | | | | | |
| Low | Ref | Ref | Ref | Ref | Ref | Ref | Ref | Ref | Ref | Ref |
| Middle | -1.88 (-4.93-1.17) | -2.81 (-6.16-0.54) | -0.34 (-1.58-0.91) | -0.28 (-1.70-1.14) | 10.26 (2.50-18.03) | 7.58 (-1.53-16.69) | 1.58 (-0.09-3.25) | 0.62 (-1.22-3.46) | 3.07 (0.83-11.40) | 0.83 (0.14-4.90) |
| High | -1.58 (-5.90-2.74) | -4.30 (-9.51-0.91) | 0.13 (-1.63-1.90) | 0.11 (-2.10-2.31) | 6.12 (-4.87-17.11) | 2.61 (-11.55-16.76) | 2.57 (0.20-4.93) | 1.40 (-1.46-4.25) | 2.95 (0.56-15.68) | 0.38 (0.04-4.17) |
| ***p-value*** | ***0.461*** | ***0.178*** | ***0.801*** | ***0.862*** | ***0.036*** | ***0.212*** | ***0.058*** | ***0.617*** | ***0.226*** | ***0.671*** |
| R^2^ | 0.227 | 0.343 | 0.145 | 0.220 | 0.175 | 0.202 | 0.216 | 0.333 | 0.146 | 0.326 |
| Coefficients (ß) from linear regressions and odds ratios (OR) from logistic regressions and their 95% confidence interval (CI).  All linear trends are shown in color. Light green indicates a trend in favor of the hypothesis, with dark green being significant at p-value < 0.05.  p-values from likelihood ratio tests  Model 2: adjusted for age, lesion level, completeness of injury, years since injury, etiology, and mobility  Model 3: adjusted for age, lesion level, completeness of injury, years since injury, etiology, mobility, and all SES indicators (displayed as Model 2 in Table 4)  Unadjusted results are displayed in Model 1 of Table 4.  Abbreviation: *Ref* Reference category | | | | | | | | | | |
